# Spondylodiscite tuberculeuse compliquée d’une ischémie aigue des membres inférieurs - à propos d’un cas

**Published:** 2011-04-22

**Authors:** Abdelkarim Shimi, Nawfal Houari, Mustapha Harrandou, Mohamed Khatouf, Nabil Kanjaa

**Affiliations:** 1 Service d’Anesthésie Réanimation polyvalente, CHU Hassan II, Fès, Maroc

**Keywords:** Thrombose, artère, tuberculose, ischémie

## Abstract

Les complications thromboemboliques associées à l’infection par *Mycobacterium tuberculosis* ont été rapportées dans la littérature et ont eu lieu dans 1,5 à 3,4% de l’infection tuberculeuse. Nous rapportons le cas d’une fillette âgée de 4ans suivie pour spondylodiscite tuberculeuse, admise dans notre formation pour prise en charge d’une ischémie aigue des deux membres inférieurs consommée, ayant nécessité une amputation transtibiale d’un côté et trans-fémorale du coté contro-latéral. Nous discutons les aspects cliniques, physiopathologiques et le lien de causalité.

## Introduction

Les complications vasculaires associées à l’infection par *Mycobacterium tuberculosis* ont été rapportées dans la littérature et ont eu lieu dans 1,5 à 3,4% de l’infection tuberculeuse [1-2]. L’ischémie aigue des membres inférieurs peut se voir dans de nombreuses situations, mais son association à une tuberculose n’a jamais été décrite à notre connaissance. Nous rapportons le cas d’une fillette âgée de 4ans suivie pour spondylodiscite tuberculeuse, admise dans notre formation pour prise en charge d’une ischémie aigue des deux membres inférieurs.

## Patient et observation

Fillette A. N. âgée de 4ans, ayant comme antécédents un père traité pour tuberculose pulmonaire à microscopie positive mis sous 2SRHZ/4RH déclaré guéri. Suivie depuis 2 mois pour spondylodiscite tuberculeuse mise sous 2SRHZ/7RH.

La patiente a présenté après 1 mois de traitement une cyanose des orteils des 2 membres inférieurs ayant évolué en quelques jours vers la gangrène des deux membres inférieurs.

**Figure 1:**
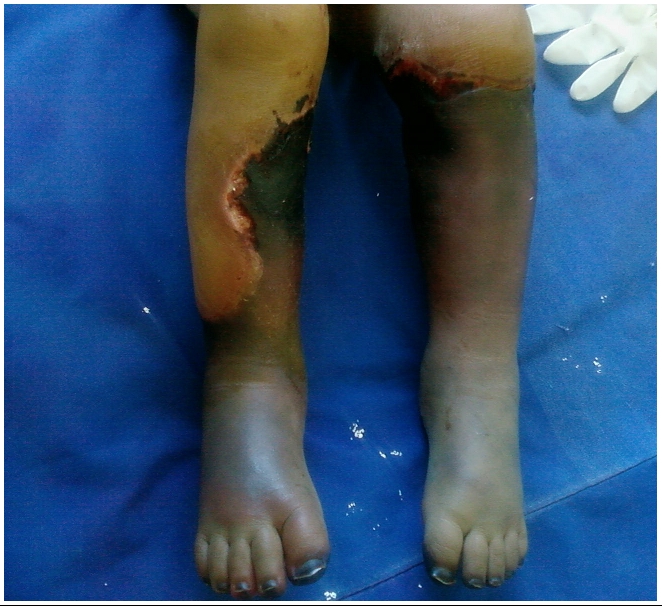
Ischémie des deus membres inférieurs chez un patient Marocain de 4 ans prérieus chez un patient Marocain de 4 ans présentant une spondylodiscite tuberculeuse compliquéle d’une ischémie aigue des members inférieurs

**Figure 2:**
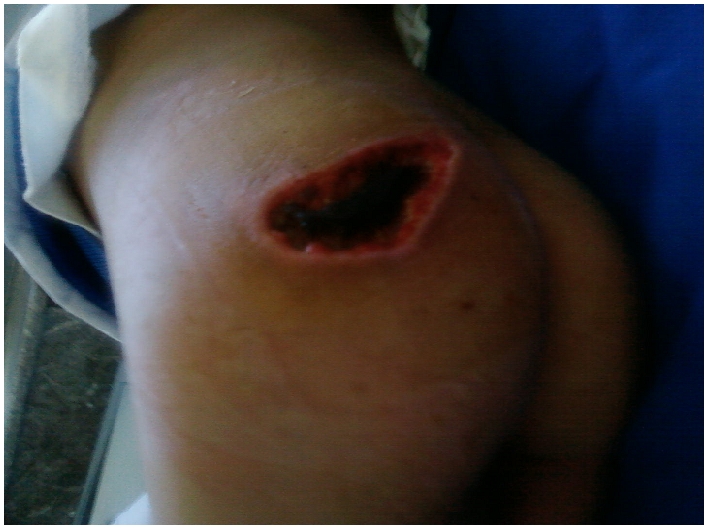
Nécrose cutanée fessière un patient Marocain de 4 ans présentant une spondylodiscite tuberculeuse compliquée d’une ischémie aigue des membres inférieurs

L’examen à l’admission a trouvé un enfant en assez bon état général, apyrétique une fréquence cardiaque à 100 battement/min. Une gangrène sèche des deux membres inférieures arrivant jusqu’au genou à gauche et intéressant la face interne de la jambe droite (Figure 1), des zones de nécrose cutanée fessière et en regard du grand trochanter gauche et de l’épine iliaque antero-supérieur gauche (Figure 2).

Le bilan biologique avait montré un taux normal d’hémoglobine et de plaquettes, une glycémie, du fibrinogène, une protéine C avec un taux de prothrombine normaux. La radiographie du thorax n’a pas montré de lésions parenchymateuses et l’électrocardiogramme était normal.

La patiente a bénéficié d’une amputation trans-tibiale à droite et trans-fémorale à gauche. Les suites opératoires ont été simples avec une bonne évolution des lésions. La patiente a été déclarée guérie en fin du traitement.

## Discussion

L’état d’hypercoagulabilité dans la tuberculose a été rarement décrit dans la littérature. L’association de la tuberculose à la thrombose veineuse profonde (TVP) [3,4], et à la thrombose veineuse corticale (CVT) [3,5,6] a été rarement signalée. Cependant l’association à une thrombose artérielle n’a été décrite qu’une seule fois dans la littérature [7]. Dans notre cas, les troubles de la coagulation ont été manifestés sous forme d’une ischémie aigue des deux membres inférieurs associée à des zones de nécrose cutanée secondaires à une hypo perfusion périphérique. Les facteurs généralement associés à la pathogenèse de la thrombose sont: l’altération de la paroi vasculaire, la modification dans les constituants du sang et le ralentissement circulatoire sanguin.

La TB disséminée peut induire au niveau du sang périphérique une Activation des cellules mononuclées, et l’interaction de ces cellules activées avec les produits de mycobactéries induit une synthèse accrue de facteur de nécrose tumorale- alpha et l’interleukine-6 [3,8,9].

La tuberculose a plusieurs mécanismes qui peuvent induire un état d’hypercoagulabilité pouvant entraîner des complications thrombo-emboliques. Différentes études ont conclu que le taux de fibrinogène plasmatique élevé, avec des facultés affaiblies de fibrinolyse associée à une diminution de la thrombine III, protéine C et l’agrégation plaquettaire semblent induire un état d’hypercoagulabilité favorisant le développement de la thrombose veineuse profonde dans la tuberculose pulmonaire [1,4,10].

Certains auteurs ont mentionnés la fréquence élevée des anticorps antiphospholipides détectés dans la tuberculose, et la relation éventuelle entre ces derniers et la protéine S. Bien que les études sur l’activité de la prothrombine dans la tuberculose ne soient pas nombreuses, il semble que hypoprothrombinémie plutôt que l’hyperactivité de prothrombine existe dans nombre appréciable de cas. Différentes études indiquent que le déficit en prothrombine survient chez environ un tiers des patients atteints de tuberculose [1,11,12].

Les cytokines, par leur caractère pro- inflammatoire, vont activer l’intima vasculaire et rendre l’endothélium thrombogène. Ils vont induire aussi une stimulation de la synthèse hépatique des protéines de la coagulation [3,13]. Ces risques d’hypercoagulabilité sont majorés par L’immobilité et l’alitement en raison de la morbidité causée par la maladie.

A l’heure actuelle, aucune incidence de thrombose artérielle n’a été rapportée, un seul cas de thrombose de l’artère axillaire associée à une tuberculose pulmonaire a été rapporté chez un patient de 45ans. Et si L’incidence des TVP cliniquement apparente rapportée a été de 3% à 4% en cas de tuberculose pulmonaire [3,14], Le taux e réel est probablement plus élevé, en raison du caractère asymptomatique de ces atteintes. Malgré que la tuberculose soit largement répandue au Maroc, les données sur l’association avec un état d’hypercoagulabilité font défaut, reflétant peut-être un manque de sensibilisation de cette association.

Bien que ces données aient été avancées, le vrai mécanisme physiopathologique n’est pas encore clair et n’est pas bien élucidé. D’autres médiateurs de l’inflammation pourraient être impliqués dans la thrombose profonde et seuls les essais cliniques peuvent répondre à ces questions. Le recours à l’anticoagulation peut être indiqué d’emblée dans certaines formes sévères de tuberculose.

## Conclusion

Bien que le l’épidémiologie et l’histoire naturelle de la tuberculose ont été abondamment décrits, un état d’hypercoagulabilité n’a été que rarement souligné. Le manque de sensibilisation concernant cette association est peut-être responsable de sa méconnaissance. Une surveillance étroite du taux de prothrombine et au besoin une anti coagulation s’impose surtout lorsque la rifampicine est associée.

## Conflits d’intérêts

Les auteurs ne déclarent aucun conflit d’intérêt.

## Contribution des auteurs

Tous les auteurs ont contribué à l’élaboration de ce travail.
